# Static and dynamic postural control deficits in aging fragile X mental retardation 1 (*FMR1*) gene premutation carriers

**DOI:** 10.1186/s11689-018-9261-x

**Published:** 2019-01-21

**Authors:** Zheng Wang, Pravin Khemani, Lauren M. Schmitt, Su Lui, Matthew W. Mosconi

**Affiliations:** 1 Department of Occupational Therapy, University of Florida, Gainesville, FL 32611 USA; 2 Department of Neurology, Swedish Neuroscience Institute, Seattle, WA 98121 USA; 3 Division of Developmental and Behavioral Pediatrics, Cincinnati Children’s Hospital Medical Center, Cincinnati, OH 45229 USA; 4 Huaxi Magnetic Resonance Research Center (HMRRC), Department of Radiology, West China Hospital of Sichuan University, Chengdu, 610041 Sichuan China; 5 Schiefelbusch Institute for Life Span Studies, University of Kansas, Lawrence, KS 66045 USA; 6 Clinical Child Psychology Program, University of Kansas, Lawrence, KS 66045 USA; 7 Kansas Center for Autism Research and Training (K-CART), University of Kansas, Lawrence, KS 66045 USA; 8 University of Florida, 1225 Center Drive, PO Box 100164, Gainesville, FL 326100164 USA

**Keywords:** Fragile X mental retardation 1 (*FMR1*) gene, *FMR1*gene premutation allele, Fragile X-associated tremor/ataxia syndrome (FXTAS), Postural control, Cerebellum

## Abstract

**Background:**

Individuals with premutation alleles of the fragile X mental retardation 1 (*FMR1*) gene are at risk of developing fragile X-associated tremor/ataxia syndrome (FXTAS) during aging. Characterization of motor issues associated with aging in *FMR1* premutation carriers is needed to determine neurodegenerative processes and establish new biobehavioral indicators to help identify individuals at greatest risk of developing FXTAS.

**Methods:**

We examined postural stability in 18 premutation carriers ages 46–77 years and 14 age-matched healthy controls. Participants completed a test of static stance and two tests of dynamic postural sway on a force platform to quantify postural variability and complexity. CGG repeat length was measured for each premutation carrier, and MRI and neurological evaluations were conducted to identify carriers who currently met criteria for FXTAS. Of the 18 premutation carriers, seven met criteria for definite/probable FXTAS (FXTAS+), seven showed no MRI or neurological signs of FXTAS (FXTAS−), and four were inconclusive due to insufficient data.

**Results:**

Compared to controls, premutation carriers showed increased center of pressure (COP) variability in the mediolateral (COP_ML_) direction during static stance and reduced COP variability in the anterior-posterior (COP_AP_) direction during dynamic AP sway. They also showed reductions in COP_ML_ complexity during each postural condition. FXTAS+ individuals showed reduced COP_AP_ variability compared to FXTAS− carriers and healthy controls during dynamic AP sway. Across all carriers, increased sway variability during static stance and decreased sway variability in target directions during dynamic sways were associated with greater CGG repeat length and more severe neurologically rated posture and gait abnormalities.

**Conclusion:**

Our findings indicate that aging *FMR1* premutation carriers show static and dynamic postural control deficits relative to healthy controls implicating degenerative processes of spinocerebellar and cerebellar-brainstem circuits that may be independent of or precede the onset of FXTAS. Our finding that FXTAS+ and FXTAS− premutation carriers differed on their level of intentional AP sway suggests that neural mechanisms of dynamic postural control may be differentially impacted in patients with FXTAS, and its measurement may be useful for rapidly and precisely identifying disease presence and onset.

**Electronic supplementary material:**

The online version of this article (10.1186/s11689-018-9261-x) contains supplementary material, which is available to authorized users.

## Background

Mutations of the fragile X mental retardation 1 (*FMR1*) gene involving > 200 cytosine-guanine-guanine (CGG) repeat expansions in the 5′-untranslated region cause fragile X syndrome is the leading inherited cause of intellectual disability. *FMR1* premutations characterized by 55–200 CGG repeats are associated with subclinical psychiatric, cognitive, and motor issues [1, 2]. Approximately one third of aging premutation carriers also develop fragile X-associated tremor/ataxia syndrome (FXTAS), a progressive neurodegenerative disorder characterized by kinetic tremor, gait ataxia, and Parkinsonism and involves neurodegenerative processes of spinocerebellar and cerebellar-brainstem circuits [1–4]. Penetrance of FXTAS among premutation carriers increases with age, and the onset of motor deficits typically occurs after 50 years of age with subsequent rapid neurological and cognitive decline [1].

Increased CGG repeat length is associated with increased risk of developing FXTAS [4–8]. Cerebellar lesions and cerebral atrophy are common [3, 4] and serve as part of the diagnostic criteria for FXTAS [1, 2, 9]. However, FXTAS often is misdiagnosed due to its clinical overlap with other neurodegenerative disorders (e.g., Parkinson’s disease), especially early in its course [10, 11]. Precise approaches for quantifying neurodegenerative processes associated with *FMR1* premutations across behavioral, neural, and genetic levels are needed to advance our understanding of the cause of the disease, identify prodromal signs, and monitor disease progression and treatment outcomes.

Spinocerebellar and cerebellar-brainstem function often is measured behaviorally using tests of static postural control during which participants stand as still as possible, and dynamic postural control in which individuals initiate continuous body sway along an axis. Paramedian cerebellar lobules including the vermis receive afferent input both from motor and posterior parietal cortices as well as direct innervation from the spinal cord [12]. Spinocerebellar inputs provide rapid proprioceptive feedback information that can be integrated with somatosensory, visual and vestibular feedback in cerebellar-cortical networks to maintain postural stability. Tests of static and dynamic stances have been used to index cerebellar dysfunctions in individuals with Friedreich’s ataxia [13], psychiatric disorders involving cerebellar-brainstem dysfunction [14], and movement disorders such as Parkinson’s disease [15]. In the present study, we investigated static and dynamic postural control in aging *FMR1* premutation carriers in order to characterize cerebellar-dependent motor processes.

Multiple prior studies have examined postural control in aging *FMR1* premutation carriers, but these studies have suggested that postural control is relatively intact in carriers who showed no signs of FXTAS [5, 10, 16]. Importantly, each prior study of premutation carriers quantified postural control using individuals’ postural sway area, which is an aggregate measure of body sway in both anterior-posterior (AP) and mediolateral (ML) directions. As increased sway variability in ML directions predicts the risk of fall in older adults [17–19] and is used to monitor the progression of cerebellar ataxia [13] and Parkinson’s disease [15, 20], precise quantification of postural sway in ML directions may be more sensitive to atypical neurodegenerative processes. Analyses separating center of pressure (COP) variability in ML and AP directions are needed to determine if postural control mechanisms are disrupted during aging in *FMR1* premutation carriers.

Assessments of dynamic postural sways in which participants continuously move their body along an axis (e.g., AP or ML) provide important information about individuals’ ability to reactively refine postural sway amplitude and velocity to maintain balance. During dynamic sway, gravitational torque increases due to increases in sway amplitude in the target direction. Increased demand to reverse postural sway acceleration prior to the body’s center of mass approaching the base of support boundary makes dynamic sway more challenging to control than static stance [21, 22]. In contrast to static stance, postural instability during dynamic stance is reflected by COP variability *reductions* in target directions that may compensate for reduced postural control as individuals attempt to avoid moving their body’s center of mass close to their base of support boundary [23]. No known studies have examined dynamic postural control in aging *FMR1* premutation carriers.

Measurement of non-linear time-dependent properties of individuals’ postural sway also may provide sensitive indices of the integrity of the postural control system [24]. Postural instability can be examined by quantifying the complexity of the long-range correlation of the COP time series along multiple temporal scales using the measure of detrended fluctuation analysis (DFA) [25, 26]. DFA assumes that postural sway involves a combination of deterministic and stochastic process [25–27]. Deterministic processes represent a “stable” state allowing individuals to sway within their base of support in a predictable manner (i.e., the regularity of postural sway). Stochastic processes reflect the integration of individuals’ internal state (e.g., proprioception) and processing of external feedback (e.g., visual, vestibular, and somatosensory) which affords flexibility in controlling postural sway relative to task demands (i.e., the complexity of the COP time series). Neurodegeneration of the cerebellum is associated with a reduction in the ability to integrate these spontaneous processes and a resulting reduction of COP complexity [14, 26]. Consistent with this idea, reduced postural sway complexity during static stance has been documented in studies of aging [26], cerebellar atrophy [14], and Parkinson’s disease [28]. Yet, the extent to which COP complexity is affected in *FMR1* premutation carriers remains unknown.

In the present study of *FMR1* premutation carriers, we examined postural control variability and complexity in both AP and ML directions across static and dynamic stances. All premutation carriers completed neurological testing to determine whether they showed clinical or radiological signs of FXTAS**.** We hypothesized that *FMR1* premutation carriers would show increased COP variability during static stance compared to healthy aging controls, with the effect more pronounced in the ML direction. During dynamic postural sways, we predicted that premutation carriers would show reduced COP variability in target directions compared to controls. For all standing postures, we hypothesized reduced COP complexity in individuals with *FMR1* premutations compared to controls. We also predicted that the severity of postural sway deficits in premutation carriers would be related to increased CGG repeats.

Based on the neurological and MRI testing, we also characterized premutation carriers as either having probable/definite (FXTAS+) or no signs of FXTAS (FXTAS−). We expected that the FXTAS+ subgroup would show reduced postural control relative to FXTAS− carriers and controls.

## Methods

### Participants

Eighteen *FMR1* premutation carriers were identified through our fragile X clinics and postings on local and national fragile X association listservs. Fourteen controls matched on age and sex were recruited through community advertisements. During an initial screening interview, no premutation carriers reported any issues of tremor or ataxia. They completed genetic testing to quantify CGG repeat length, a T2-weighted MRI scan and a structured neurological evaluation conducted by a clinical neurologist (PK) using the International Cooperative Ataxia Rating Scale (ICARS) [29]. All participants completed the abbreviated battery of the Stanford-Binet Intelligence Scales, Fifth Edition [30] to characterize cognitive abilities, including nonverbal fluid reasoning and verbal knowledge (Table 1). Participants then completed tests of static and dynamic postural stances. All study procedures were approved by the local IRB.

**Table 1:** Demographics characteristics, and cognitive and clinical scores (mean ± SD) of *FMR1* gene premutation carriers and healthy aging control participants

Characteristics	Premutation carriers (*n* = 18)	Controls (*n* = 14)	F	*p*
Age (year)	61.89 (7.40)	57.64 (8.92)	1.267	0.269
Height (cm)	165.95 (8.01)	167.28 (8.94)	0.196	0.661
Weight (kg)	90.31 (20.19)	79.92 (20.17)	2.087	0.159
Male (*N*)^a^	6	6	0.305	0.581
Full-scale IQ	102 (12)	112 (15)	2.685	0.112
ICARS speech	0.19 (0.54)	**–**	**–**	**–**
ICARS kinetic	1.50 (2.03)	**–**	**–**	**–**
ICARS oculomotor	0.75 (1.00)	**–**	**–**	**–**
ICARS posture and gait	3.00 (2.28)	**–**	**–**	**–**
ICARS total	5.44 (4.98)	**–**	**–**	**–**

International Cooperative Ataxia Rating Scale (ICARS) scores only available for 16 premutation carriers

^a^Chi-square statistics

Both *FMR1* premutation carriers and control participants were excluded if they reported lower extremity orthopedic surgery within the past year, or any musculoskeletal disorder that could potentially cause atypical postural or gait functioning, or a history of medications known to affect motor functioning [31]. Eight participants (seven premutation carriers, one control) reported being on medication within 48 h of testing, including antidepressants (selective serotonin reuptake inhibitors: wo premutation carriers, one control who reported taking medication for premenstrual syndrome; serotonin-norepinephrine reuptake inhibitors: two premutation carriers), sedatives/hypnotics (benzodiazepine anxiolytic: one premutation carrier; nonbenzodiazepine anxiolytic: one premutation carrier), synthroids (three premutation carriers), or a mood stabilizer (one premutation carrier).

### Procedure and approach

#### CGG repeat length

*FMR1* CGG repeat length was quantified for all premutation carriers. Molecular testing was conducted at Dr. Berry-Kravis’ Molecular Diagnostic Laboratory at Rush University. Genomic DNA was isolated from peripheral blood leukocytes samples. The *FMR1* polymerase chain reaction (PCR) test with quantification of allele-specific CGG repeat length was performed using commercially available kits (Asuragen, Inc., Austin, TX).

#### T2-weighted magnetic resonance imaging (MRI) scan

*FMR1* premutation carriers underwent a T2-weighted MRI scan (repetition time = 6350 msec; echo time = 100 msec; flip angle = 120°; field of view = 256 × 156 × 256 mm^3^; 78 axial slices; voxel size = 1 mm^2^ × 2 mm; no gap) to test for the presence of hyperintensities within the middle cerebellar peduncle (i.e., the MCP sign), cerebral atrophy, or other cerebral or cerebellar-brainstem alterations associated with FXTAS [2–4]. T2-weighted scans were analyzed by a trained neuroradiologist (SL) with expertise in diseases of aging.

#### Neurological examination

*FMR1* premutation carriers completed a structured neurological exam administered by a neurologist (PK) with expertise in ataxia and movement disorders in aging. This exam included evaluations of movement and gait as well as administration of the ICARS. The ICARS is comprised of 19 sections examining postural and gait disturbances, ataxia, dysarthria, and oculomotor functions. Higher scores indicate a higher level of cerebellar ataxia. The ICARS has been validated previously for diagnosis in patients with focal cerebellar lesions [32] and Friedrich’s ataxia [33].

#### Postural control assessments

Postural stability was assessed using an AMTI (American Mechanical Technology, Inc., Watertown, MA) AccuGait strain gauge force platform (size 49.78 × 49.78 cm) with a sampling rate of 1000 Hz. All participants completed tests of static and dynamic stances by standing on the platform with bare feet shoulder-width apart and arms resting at their sides. Participants’ foot positions were outlined on a piece of tracing paper placed on top of the force platform prior to the first trial to ensure consistent placement and orientation of the feet during each trial. During the static stance test, participants were instructed to stand as still as possible for three 30-s trials. During dynamic stance tests, participants completed three 30-s trials for each of two different self-initiated postural sways—AP and ML. For each dynamic stance condition, participants were instructed to sway continuously in the target direction at a comfortable speed and amplitude without raising their toes or heels. Each stance trial was followed by 30 s of rest. Nine trials (three conditions × three trials) were examined in total. Order of administration of the static and dynamic stance tests and the two directions of the dynamic stance test were counterbalanced across participants.

The force and moment data collected from the force plate were down sampled to 200 Hz and low pass filtered using a fourth-order double pass Butterworth filter with a cutoff frequency of 6 Hz in Matlab 2017a (MathWorks, Inc., Natick, MA). The COP time series were derived from the force and moment data for each standing posture [22]. The variability of individuals’ postural sway was quantified using COP standard deviation in both the AP (COP_AP_) and ML (COP_ML_) directions as we did previously [34].

The complexity of individuals’ postural sway in both directions was quantified using the *α* exponent of DFA [25, 26]. DFA is a non-linear measurement quantifying the pattern of variation of a time series across multiple time scales [25, 26]. Its computation is based on the assumption that variations present in a system due to intrinsic dynamics exhibit fractal properties of long-range correlations (see Appendix for the detailed algorithm). In brief, the *α* exponent of DFA varies between 0 and 2 (i.e., 0 < *α* < 2) including four ranges of values separated at 0.5, 1, 1.5, and 2. When 0 < *α* < 0.5 or 1 < *α* < 1.5, the time series is anti-correlated with a smaller *α* representing increased anti-correlation and complexity of the signal. When 0.5 < *α* < 1 or 1.5 < *α* < 2, increased alpha represents increased long-range correlation and reduced complexity of the time series. The COP time series consist of 6000 data points (30 s × 200 data points/s), which has been shown to be sufficient for analyses of DFA [35].

### Statistical analyses

The COP standard deviation and *α* exponent of DFA were averaged across trials and compared between groups (*FMR1* premutation carriers vs. controls) using separate repeated measures ANOVAs including stance condition (static vs. AP sway vs. ML sway) and COP direction (AP vs. ML) as within subjects factors. The Greenhouse–Geisser estimate was used to provide a conservative test of ANOVA main and interaction effects for all repeated measure ANOVAs in which Mauchly’s test indicated a violation of sphericity. Statistically significant interaction effects were probed using Bonferroni corrected post-hoc analyses. All assumptions of normality and homogeneity of variance were verified for each measure of postural control.

Pearson correlations were conducted to determine the inter-relationships of posture variables found to be significantly different between groups in our main analyses and CGG repeat length. Due to the non-normal distributions of ICARS scores, Spearman correlations were applied to examine the relationships between COP-dependent variables and ICARS posture and gait subscale and total scores. The relationships between CGG repeat length and each ICARS subscale score and ICARS total scores also were examined using Spearman correlations. Correlations were interpreted as significant if ∣r∣ > 0.5.

Based on the MRI and ICARS evaluations, premutation carriers were identified as having (FXTAS+) or not having FXTAS (FXTAS−) according to published clinical criteria [1, 2, 9]. FXTAS+ individuals (*n* = 7) included premutation carriers with one major MRI sign plus one major neurological sign (definite FXTAS) and those with either one major MRI sign plus one minor neurological sign or those with two major neurological signs (probable FXTAS). FXTAS− individuals included premutation carriers with no MRI or clinical signs of FXTAS (*N* = 7). There was no difference between FXTAS+ and FXTAS− subgroups in the number of medications reported. Four premutation carriers who failed to complete the neurological evaluation due to scheduling issues (*N* = 2) or MRI scan due to claustrophobia (*N* = 2) were excluded from analyses comparing FXTAS+ individuals, FXTAS− individuals, and controls. Separate Kruskal–Wallis tests were performed on COP standard deviation and *α* exponent of DFA measures to examine group differences (FXTAS+ vs. FXTAS− vs. healthy controls). One-way ANOVAs also were performed to compare age and CGG repeat length between FXTAS+ and FXTAS− individuals. All results were interpreted as significant if *p* < 0.05.

## Results

### Postural sway variability in *FMR1* premutation carriers and healthy controls

**Fig.1:**
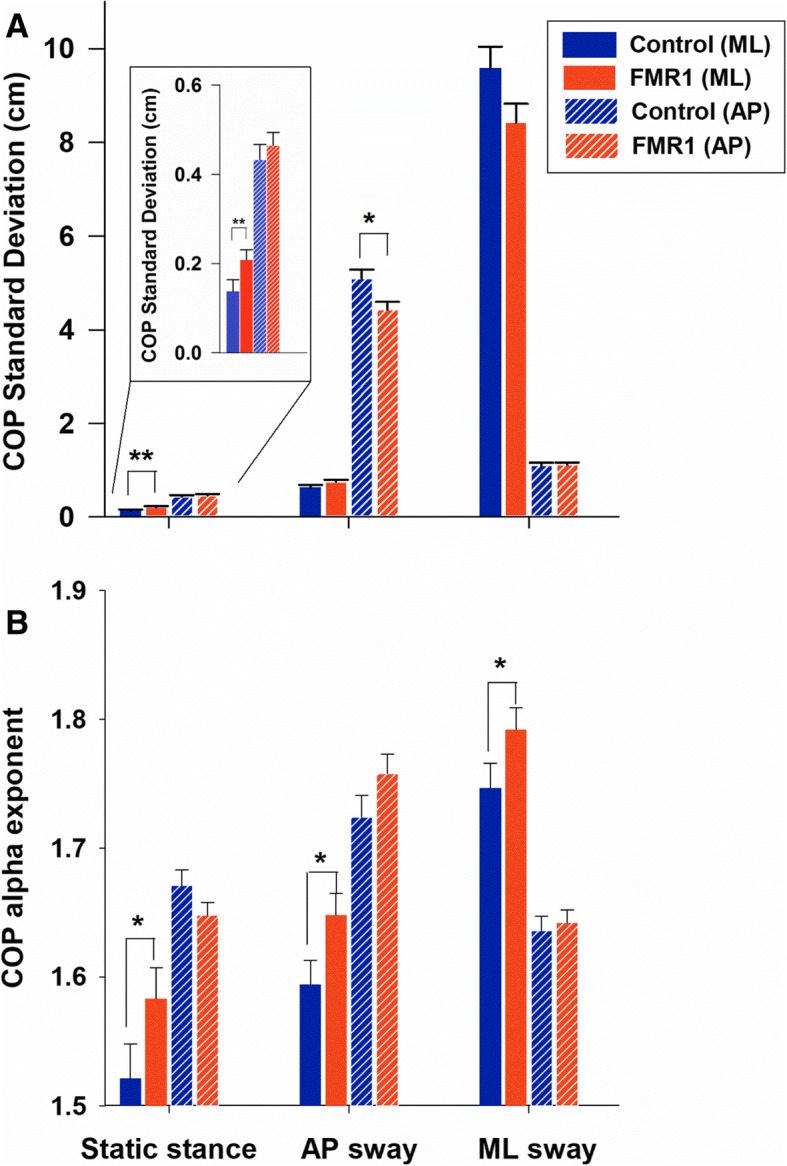
**a** Center of pressure (COP) standard deviation in the anterior-posterior (AP) and mediolateral (ML) directions. **b** The *α* exponent of detrended fluctuation analysis (DFA) of COP time series in both directions are shown as a function of standing condition. *FMR1* stands for *FMR1* premutation carriers. Between-group differences are marked as * at 0.05 level and ** at 0.01 level. Error bars represent standard error

COP standard deviation was greater during dynamic stances compared to static stance (stance condition main effect: F_1.304, 28.991_ = 643.203, *p* < 0.001; Fig. 1a). COP standard deviation was greater in the AP than in the ML direction during static stance, whereas it was greater in the target direction during dynamic stances (stance condition × direction interaction effect: F_1.115, 28.991_ = 933.867, *p* < 0.001). The interaction effect of stance condition, direction, and group was significant (F_1.115, 28.991_ = 6.082, *p* = 0.017). Relative to controls, *FMR1* premutation carriers showed increased COP_ML_ standard deviation during static stance (*FMR1* − controls = 0.071 cm, SE = 0.034 cm with F_1,26_ = 4.334, *p* = 0.047). During dynamic AP postural sway, premutation carriers showed lower COP standard deviation in the target directions compared to healthy controls (*FMR1* − controls = − 0.668 cm, SE = 0.245 cm with F_1,26_ = 7.435, *p* = 0.011). Premutation carriers also showed less COP sway in the target direction during the ML condition, although this effect did not reach statistical significance (*FMR1* − controls = − 1.157 cm, SE = 0.604 cm with F_1,26_ = 3.663, *p* = 0.067).

### Postural sway complexity in *FMR1* premutation carriers and healthy controls

The *α* exponent of COP_AP_ and COP_ML_ showed significant increases during dynamic stances compared to the static stance condition (stance condition main effect: F_1.645, 54,837_ = 73.166, *p* < 0.001; Fig. 1b). The *α* exponent was greater in the AP direction compared to the ML direction during static stance (stance condition × direction interaction effect: F_1.891, 54,837_ = 121.022, *p* < 0.001; static stance: COP_AP_− COP_ML_ = 0.108, SE = 0.016 with *p* < 0.001), while it was greater in the target directions during both dynamic stances (dynamic AP sway: COP_AP_ − COP_ML_ = 0.120, SE = 0.012 with F_1, 26_ = 104. 534, *p* < 0.001; dynamic ML sway: COP_ML_ − COP_AP_ = 0.131, SE = 0.013 with F_1, 26_ = 111.511 *p* < 0.001). *FMR1* premutation carriers showed a greater *α* exponent in the ML direction than control participants across all standing conditions reflecting reduced complexity of their COP time series (stance condition × direction × group interaction: F_1, 58_ = 7.572, *p* = 0.010; static stance: *FMR1*-controls = 0.062, SE = 0.027, F_1, 26_ = 16.622, *p* < 0.001; dynamic AP sway: *FMR1*-controls = 0.054, SE = 0.019, F_1, 26_ = 24.905, *p* < 0.001; dynamic ML sway: *FMR1*-controls = 0.045, SE = 0.024, F_1, 26_ = 4.308, *p* < 0.05).

### Postural sway in FXTAS+ and FXTAS− carriers

Table 2 summarizes CGG repeat length, radiological and neurological results for each *FMR1* premutation carrier. Results from the Kruskal-Wallis test showed a group main effect of COP_AP_ standard deviation (*χ*^2^(2) = 12.112, *p* = .002) during dynamic AP sway (Fig. 3) characterized by reduced COP_AP_ variability in FXTAS+ individuals compared to FXTAS− individuals (*χ*^2^(1) = 7.547, *p* = .006) and control participants (*χ*^2^(1) = 10.776, *p* = .001). No differences in COP variability for any direction or stance were found between FXTAS− and healthy control participants (*χ*^2^(1) = .050, *p* = .823). One-way ANOVAs performed on age (F _1,12_ = .733, *p* = .409) and CGG repeat length (F _1,12_ = 1.612, *p* = .228) showed no difference between FXTAS+ and FXTAS− individuals.

**Table 2:** CGG repeat length, international cooperative ataxia rating scale (ICARS) scores, radiological and neurological evaluations, and clinical classification for each individual *FMR1* gene premutation carriers

ID	CGG repeats	ICARS					T2 scan	Neurological exam	Clinical classification
		Speech	Kinetic	Oculomotor	Gait and posture	Total			
1	87	0	0	1	1	2	Generalized WM lesion, cerebral atropy type 1	No gait ataxia, no tremor	No FXTAS^a^
2	102	0	0	1	2	3	Generalized white matter lesion; cerebral atrophy type 2	No gait ataxia, no tremor	No FXTAS
3	58	0	0	1	3	4	(−)	No gait ataxia, no tremor	No FXTAS
4	58	0	0	0	2	2	(−)	Tremor, no gait ataxia	No FXTAS
5	62	0	1	0	1	2	Mild white matter lesion, dot-like white matter hyperintensity	Tremor, no gait ataxia	No FXTAS
6	68	1	0	0	0	1	Mild white matter lesion, dot-like white matter hyperintensity	No gait ataxia, no tremor	No FXTAS
7	80	0	0	0	1	1	Mild white matter lesion, dot-like white matter hyperintensity, cerebral atrophy type 2	No gait ataxia, no tremor	No FXTAS
8	99	0	2	0	5	7	(−)	Mild gait ataxia, mild tremor	Probable FXTAS
9	107	0	1	2	5	8		Gait ataxia, tremor	Probable FXTAS
10	81	0	1	0	4	5	Cerebral atrophy type 1	Gait ataxia, tremor	Probable FXTAS
11	75	2	7	3	7	19		Gait ataxia, tremor	Probable FXTAS
12	58	0	3	0	5	8		Gait ataxia, tremor	Probable FXTAS
13	85	1	2	2	7	12	MCP sign, generalized white matter lesion; cerebral atrophy type 3	Gait ataxia, tremor	FXTAS
14	93	0	5	0	3	8	Suspected MCP sign, 4th ventricle widening, cerebral atrophy type 1, cerebellum and brainstem atrophy	Tremor, no gait ataxia	FXTAS
15	102	0	0	0	0	0			Inconclusive
16	90	0	2	2	2	6		Tremor, no gait ataxia	Inconclusive
17	64								Inconclusive
18	78						Mild white matter lesion, dot-like white matter hyperintensity		Inconclusive

11: CGG repeat length was identified from individual’s previous genetic exam at the Department of Human Genetics at Emory University School of Medicine

14: CGG repeat length was identified from individual’s previous genetic exam at the Center for Genetic Services in Corpus Christi in Taxes

(−) entry: no abnormality were identified

No entry: data were not collected

^a^ No FXTAS: *FMR1* gene premutation carriers who currently do not how radiological and neurological signs of FXTAS

### Clinical associations

**Fig. 2:**
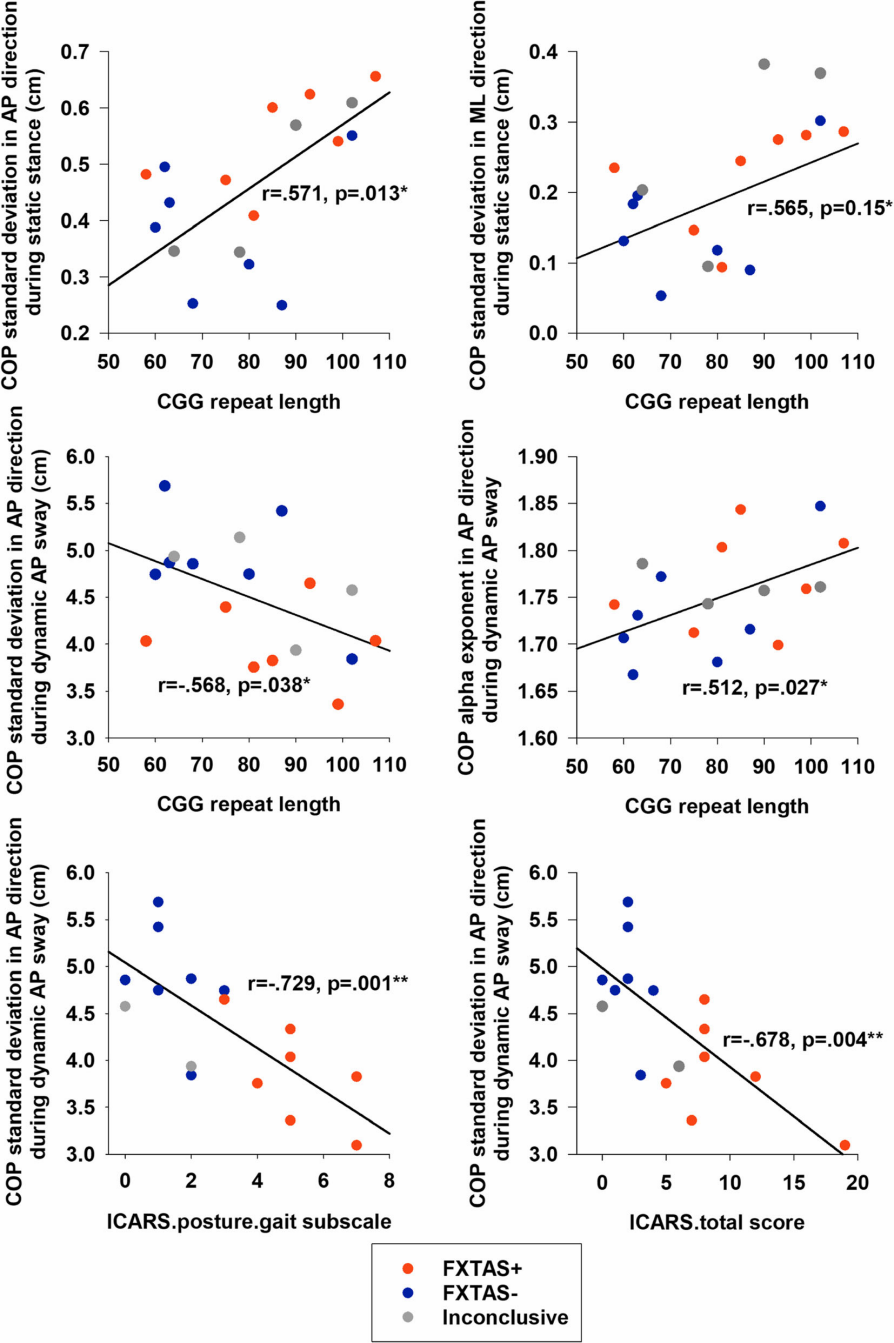
Scatter plots of significant statistical correlations presented in Table 3. Data were color-coded based on the diagnostic classification of each individual *FMR1* premutation carrier. ICARS scores were missing for two inconclusive individuals due to scheduling issues

Greater COP variability in both the AP and ML directions during static stance was associated with higher CGG repeats in *FMR1* premutation carriers (Table 3; Fig. 2). Lower sway variability in target directions during dynamic AP sway also was associated with higher CGG repeats in premutation carriers. Greater *α* exponent of DFA in target directions during dynamic AP postural sway was related to higher CGG repeats. Lower COP variability in the AP direction during dynamic AP sway was associated with higher ICARS posture and gait subscale and ICARS total scores in premutation carriers. None of the ICARS subscale scores were associated with CGG repeat length (dysarthria: *r* = − 0.090, *p* = 0.742; kinetic ataxia: *r* = 0.003, *p* = 0.991; oculomotor disorders: *r* = − 0.238, *p* = 0.374; gait and posture: *r* = 0.010, *p* = 0.972; total: *r* = 0.088, *p* = 0.745). CGG repeat length was not associate with any postural control variables or ICARS scores within the FXTAS+ or FXTAS− subgroups (Additional files 1 and 2: Tables S1 and S2**).**

**Table 3:** Correlation coefficients (*r*) between cytosine-guanine-guanine (CGG) repeat length, ICARS posture and gait subscale and total scores, and center of pressure (COP) measures of *FMR1* premutation carriers

	Static stance	Anterior-posterior (AP) sway	Mediolateral (ML) sway
CGG repeat length			
COP_AP_-SD	*.571* ^b^	*− .568* ^b^	− .229
COP_ML_-SD	*.565* ^b^	.467	− .413
COP_AP_-DFA alpha	.049	*.512* ^b^	− .262
COP_ML_-DFA alpha	.368	.138	.268
ICARS posture and gait score^a^			
COP_AP_-SD	0.329	*− 0.729* ^c^	− 0.002
COP_ML_-SD	0.181	− 0.394	− 0.042
COP_AP_-DFA alpha	0.223	0.364	− 0.417
COP_ML_-DFA alpha	0.274	0.019	0.351
ICARS total score^a^			
COP_AP_-SD	0.459	*− 0.678* ^c^	0.060
COP_ML_-SD	0.287	− 0.224	0.044
COP_AP_-DFA alpha	0.104	0.232	− 0.372
COP_ML_-DFA alpha	0.268	− 0.010	0.247

Significant values are in italics

^a^ Spearman correlation coefficient (*ρ*)

^b^ Significant at alpha level of 0.05

^c^ Significant at alpha level of 0.01

## Discussion

**Fig. 3:**
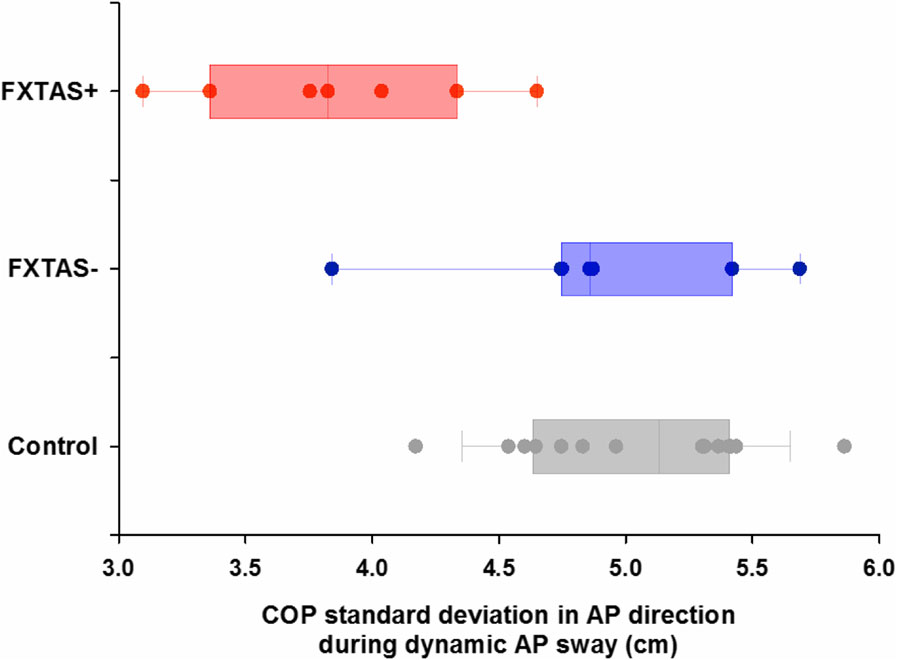
Scatter plot of COP standard deviation in AP directions during dynamic AP sway. Data are color-coded based on the diagnostic classification of *FMR1* premutation carriers and healthy controls. Box plots show (left to right) the minimum (cap), first quartile, median, third quartile, and maximum (cap) values of each group. Two data points in the control group were located outside of the 1.5× inter-quartile range

In the present study, we provide new evidence that during middle to late adulthood, *FMR1* premutation carriers show reduced postural stability that is related to larger CGG repeat expansions, and thus covaries with FXTAS disease risk [5–8]. Four key findings are highlighted. First, relative to controls, *FMR1* premutation carriers showed greater postural sway variability in the ML direction during static stance and reduced postural sway along the target direction during intentional AP sway suggesting that spinocerebellar and cerebellar-brainstem circuits supporting postural stability are disrupted in aging *FMR1* premutation carriers (Fig. 1a). Second, premutation carriers demonstrated reduced complexity of their COP_ML_ time series across all standing conditions implicating deficits in their ability to dynamically adapt to inherent postural perturbations (Fig. 1b). Third, both COP variability and complexity alterations in *FMR1* premutation carriers were associated with higher CGG repeats and ICARS-rated posture, gait, and motor deficits, suggesting that our measures may provide sensitive and highly quantifiable biologically based markers of behavioral and neurological features associated with FXTAS risk or progression in premutation carriers (Fig. 2). Last, FXTAS+ individuals showed reduced AP postural sway variability during dynamic AP trials relative to carriers without clinical signs of FXTAS (FXTAS−) and healthy controls suggesting that mechanisms supporting intentional sway may be selectively disrupted in FXTAS (Fig. 3). Our measure of dynamic AP sway thus may be useful for rapidly and precisely differentiating premutation carriers with and without FXTAS.

### Postural control deficits in *FMR1* premutation carriers during static stance

Postural control is a continuous process during which individuals actively align their body’s center of mass within their base of support area in response to inherent noise and changes in environmental (e.g., moving to a slippery surface) and task (e.g., when leaning forward to reach an object; when moving from sitting to standing) demands [21]. Increases in postural sway variability are common in aging adults and reflect neurodegenerative processes involving decreased nerve conduction velocity, deterioration of visual, vestibular and somatosensory feedback systems, reduced muscle strength, and degeneration of central modulation of motoneuron pools [21, 36]. However, more severe increases in postural sway variability and reductions in complexity can reflect pathology of the cerebellum [13], basal ganglia [15], or cortical motor areas [25]. In the context of documented reductions of cerebellar and brainstem volumes [4], deterioration of cerebellar white matter microstructure [3], and increased rates of cerebellar cellular intranuclear inclusions [37] in individuals with FXTAS, our results of greater COP_ML_ variability (Fig. 1a) and reduced COP_ML_ complexity (Fig. 1b) in premutation carriers implicate spinocerebellar and cerebellar-brainstem processes independent of or prior to the onset of FXTAS. It is possible that similar deficits are evident earlier in life in premutation carriers and thus are not reflective of neurodegenerative processes, but instead motor control issues related to the premutation allele. Although one prior study [38] indicated that postural control is relatively intact during middle adulthood in female premutation carriers, direct comparisons of younger adult premutation carriers and controls on our measures are needed to determine whether postural control deficits represent atypical neurodevelopmental or neurodegenerative processes or both.

### Postural control deficits in *FMR1* premutation carriers during dynamic postural sways

During dynamic postural sways, premutation carriers showed reduced COP variability in target directions compared to healthy aging adults. When aging individuals intentionally lean in one direction, they often present a reduced ability to approach their base of support boundaries [39]. This behavioral change during aging servers as a compensatory mechanism for individuals’ reduced ability to maintain stability or recover from shifts in their COP [21]. Our results suggest this compensatory mechanism is utilized to an even greater degree by aging individuals with *FMR1* premutation alleles as they counterbalanced their postural instability by minimizing body movements away from the neutral position during dynamic stances (Fig. 1a). Results indicating that reduced dynamic sway is associated with longer CGG repeat length (Fig. 2) implicate more severe spinocerebellar or cerebellar-brainstem dysfunctions or degeneration for those individuals with greater CGG repeats. Given that greater CGG repeat length confers increased risk for the development of FXTAS, our findings also indicate that reduced dynamic sway variability may represent an early behavioral marker of FXTAS disease risk and progression.

Pathological postural sway is characterized by increased local stability and reduced complexity of the COP time series [24]. Reduced postural sway complexity in premutation carriers implicates deficient central integration of sensory feedback processes, movement anticipation, motor planning, and systems supporting coordinated musculoskeletal execution of motor commands [25, 26]. Our findings of reduced COP_ML_ complexity ( *α* exponent of DFA measure was within the range from 1.5 to 2 indicating increased long-range correlation and reductions in COP_ML_ complexity) in premutation carriers are consistent with previous studies documenting progressive decay of lateral postural sway associated with spinocerebellar and cerebellar-brainstem circuitry decline in Friedreich’s ataxia [13]. Evidence of reductions in COP variability in target directions and in stochastic processes of lateral sway in *FMR1* premutation carriers each suggest atypical deterioration of dynamic postural control mechanisms involved in modulating center of mass movement in relation to the base of support [21, 34] .

### FXTAS specific deficits of postural control

Subgroup analyses of carriers with and without FXTAS suggested that reductions in dynamic AP sway variability are specific to FXTAS+ individuals and are largely absent in FXTAS− premutation carriers (Fig. 3). Notably, FXTAS+ and FXTAS− individuals showed minimal overlap in their level of dynamic AP sway variability suggesting this measure may provide a highly sensitive and specific index of FXTAS risk or progression. COP_AP_ during dynamic AP sway also was the only measure of postural control that was associated with greater CGG repeat length and ICARS posture and gait subscale and total scores (Table 3) in premutation carriers, suggesting that it represents a highly quantifiable biobehavioral indicator of the presence of or risk for FXTAS. These findings are consistent with the well-documented cerebellar pathology present in many FXTAS patients and the cerebellum’s known role in postural control [3, 4]. Our dynamic postural control tests provide significant advantages over current clinical and neurological evaluations for identifying FXTAS as they are highly precise and efficient (e.g., they require 3–5 min to administer). In combination with genetic and MRI exams, dynamic postural control variability in the AP direction may serve as a reliable marker identifying the presence of FXTAS or helping to guide clinical assessments and screening. While further testing is needed to determine both sensitivity and specificity of our measures across a larger number of aging premutation carriers and assessing their utility across male and female premutation carriers who may present with different symptoms associated with FXTAS [1, 40], our results suggest reduced dynamic AP sway may be a rapid and precise measure useful for identifying FXTAS in aging premutation carriers.

### Neurobiological mechanisms underlying postural control deficits in aging *FMR1* premutation carriers

Our results document that increased CGG repeats are associated with increased postural sway variability in both the AP and ML directions during static stance and decreased sway variability during dynamic AP sway among premutation carriers (Table 3; Fig. 2). Previous studies suggest that higher CGG repeats are associated with increased risk for and severity of FXTAS [6–8, 41]. At the molecular level, the CGG premutation allele results in increased FMR1 mRNA levels and, in some cases, decreases in fragile X mental retardation protein (FMRP) [42, 43]. Increased FMR1 mRNA is linked to a cumulative cytotoxic effect associated with intranuclear inclusions observed in neuronal and astrocytic nuclei of the cerebellum and brainstem in postmortem tissue [37, 41]. FMRP plays an important role in RNA-binding translation and channel-binding regulation at synapses [44] affecting the formation of axons, myelination [45], and dendritic maturation [46]. Reductions of FMRP in premutation carriers could disrupt the microstructural integrity of white matter in the primary fiber pathways of the cerebellum, including the superior, middle, and inferior cerebellar peduncles, as well as other large white matter fiber tracts such as the corpus callosum [3]. These cellular and brain system alterations have been documented in individuals with FXTAS, including selective degeneration of the middle cerebellar peduncle—the primary input pathway of the cerebellum [2, 4, 9]. Importantly, cerebellar inputs from neocortical regions are critical to the cerebellum’s role in integrating internal and external sensory feedback information in order to dynamically calibrate motor output. Greater motor variability [34, 47, 48] and reduced sensorimotor complexity [49] each have been demonstrated in individuals with disorders affecting the cerebellum. The postural control deficits reported here in premutation carriers thus may provide important indices of cerebellar mechanisms contributing to clinical issues in individuals with *FMR1* premutations.

### Study limitations and future directions

While the present study documents several novel findings useful for identifying pre-clinical signs of FXTAS, our results should be considered in the context of a few limitations. First, longitudinal studies are needed to determine how measures of postural variability and complexity vary across ages in premutation carriers and whether postural control issues identified in this study are evident earlier in life in premutation carriers and thus are not reflective of the aging process associated with *FMR1* premutation, but instead motor control issues related to downstream effects of premutation alleles. Second, FXTAS disproportionately affects males, and the nature of symptoms appear to vary across males and females due to X-inactivation effects [50, 51]. Future studies are needed to clarify postural control processes in larger samples of male and female aging premutation carriers and determine their course in individuals with and without FXTAS.

## Conclusions

Our results suggest that increased postural sway variability during static stance, decreased body movement in target directions during dynamic stance, and decreased lateral sway complexity are present in aging *FMR1* premutation carriers compared to controls and are associated with larger CGG repeat expansions. Importantly, we also find that FXTAS+ individuals show reduced COP_AP_ variability during dynamic sway relative to FXTAS− carriers, suggesting that reductions in the ability to control AP sway may provide a highly quantifiable and rapid biobehavioral index of FXTAS. Taken together, these results indicate that *FMR1* premutation carriers experience reduced postural control implicating cerebellar-brainstem circuits and that precision measures of postural control may provide useful indicators of FXTAS risk or progression and important markers of disease-related mechanisms.

### Additional files

Additional file 1: **Table S1.** Correlation coefficients (r) between CGG repeat length and all COP measures and ICARS subscale and total scores of FMR1 premutation carriers without FXTAS (FXTAS− subgroup, *N* = 7). (DOCX 15 kb)

Additional file 2: **Table S2.** Correlation coefficients (r) between CGG repeat length and all COP measures and ICARS subscale and total scores of FMR1 premutation carriers with FXTAS (FXTAS+ subgroup, *N* = 7). (DOCX 15 kb)

## Appendix

Detrended fluctuation analysis (DFA) is a non-linear measurement quantifying the pattern of variation of a time series across multiple time scales [17, 20]. DFA is based on the assumption that variations present in a system due to its intrinsic dynamics exhibit fractal properties of long-range correlations. To calculate the *α* exponent of DFA, we first let X_t_ represent a COP time series and then divided it into multiple non-overlapping time windows of equal length including n samples. For each time window, a local linear trend was calculated by minimizing the root mean square errors within the window and calculating Y_t_ as a series of straight lines fits for all windows of the COP time series (N/n; N stands for the total number of samples in a trial, which is 30 sec × 200 data points/ sec = 6000 data points). The root mean square deviation from Y_t_ (i.e., the fluctuation, *F* (*n*)) then was calculated using the following formula [26]:

1 $$ F(n)={\left[\frac{1}{N}\sum \limits_{t=1}^N{\left({X}_t-{Y}_t\right)}^2\right]}^{1/2} $$

This procedure was repeated over a range of different window sizes (n), and a log_10_-log_10_ coordinate of n against *F* (*n*) was constructed. A straight line on this log_10_-log_10_ graph indicates statistical self-affinity expressed as *F*(*n*) ∝ *n*^*α*^. The scaling exponent *α* is then calculated as the slope of a straight line fit to the log_10_-log_10_ graph of *n* against *F* (*n*) using least root mean square errors. In the current study, logarithmically spaced intervals of lengths n from 4 to 2000 samples were used and the slope *α* of the log_10_–log_10_ representation of *F* (*n*) versus n was estimated to fall between n = 0.02 sec and n = 10 sec. The *α* exponent varies between 0 and 2 (i.e., 0< *α* < 2). When *α* <0.5 or 1< *α*<1.5, the time series is anti-correlated with a smaller *α* representing increased anti-correlation and complexity. When 0.5 < *α*<1 or 1.5< *α*< 2, the signal is consistent with a greater *α* value representing increased long-range correlation and reduced complexity of the signal.

## Abbreviations

AP: Anterior-posterior
CGG: Cytosine-guanine-guanine
COP: Center of pressure
COP_AP_: Center of pressure in the anterior-posterior direction
COP_ML_: Center of pressure in the mediolateral direction
DFA: Detrended fluctuation analysis
FMR1 mRNA: FMR1 messenger RNA
*FMR1*: Fragile X mental retardation 1
FMRP: Fragile X mental retardation protein
FXTAS: Fragile X-associated Tremor/Ataxia syndrome
ICARS: International Cooperative Ataxia Rating Scale
ML: Mediolateral
MRI: Magnetic resonance imaging

## Glossary

Force: Force is a push or a pull on an object. If the net force on an object is not zero, then the object accelerates (or changes its velocity). Force is a vector and has both magnitude and direction. Force recorded from a force platform includes measurements in three dimensions, including anterior-posterior, mediolateral, and vertical directions. Force along the vertical direction is typically referred to as the ground reaction force.
Moment: The moment is the turning effect produced by a net force perpendicular to the point of rotation.
COP: The point location of the vertical ground reaction force vector. The COP represents a weighted average of pressures over the surface area (i.e., feet) in contact with the ground. It has been used as an indirect measure of individuals’ postural sway. The COP can be derived from the force and moment data collected from a force platform.
COP_AP_: Center of pressure time series in the anterior-posterior direction.
COP_ML_: Center of pressure time series in the mediolateral direction.
