# Supplementary material for: Static and dynamic postural control deficits in aging fragile X mental retardation 1 (FMR1) gene premutation carriers
Source: J Neurodev Disord. 2019 Jan 21;11:2. doi: 10.1186/s11689-018-9261-x (PMC6341725; doi:10.1186/s11689-018-9261-x)
Supplement: Supplementary file 1 — Table S1. Correlation coefficients (r) between CGG repeat length and all COP measures and ICARS subscale and total scores of FMR1 premutation carriers without FXTAS (FXTAS− subgroup, N = 7). (DOCX 15 kb) [file 11689_2018_9261_MOESM1_ESM.docx]

**Additional file 1: Table S1**

| Static stance COP_ML_ standard deviation | r=.41, p=.36 |
| --- | --- |
| Static stance COP_AP_ standard deviation | r=.13, p=.78 |
| Dynamic AP sway COP_ML_ standard deviation | r=.34, p=.46 |
| Dynamic AP sway COP_AP_ standard deviation | r=-.56, p=.20 |
| Dynamic ML sway COP_ML_ standard deviation | r= -.26, p=.57 |
| Dynamic ML sway COP_AP_ standard deviation | r= .08, p=.86 |
| Static stance COP_ML_ alpha exponent | r=.00, p=.99 |
| Static stance COP_AP_ alpha exponent | r=.04, p=.93 |
| Dynamic AP sway COP_ML_ alpha exponent | r=.22, p=.63 |
| Dynamic AP sway COP_AP_ alpha exponent | r=.40, p=.37 |
| Dynamic ML sway COP_ML_ alpha exponent | r=.10, p=.83 |
| Dynamic ML sway COP_AP_ alpha exponent | r=.16, p=.76 |
| ICARS dysarthria score | ρ =.00, p=1.00 |
| ICARS kinetic score | ρ =-.41, p=.36 |
| ICARS oculomotor score | ρ =.29, p=.53 |
| ICARS posture & gait score | ρ =-.26, p=.57 |
| ICARS total score | ρ =-.21, p=.66 |

**Table S1. Correlation coefficients (r) between CGG repeat length and all COP measures and ICARS subscale and total scores of FMR1 premutation carriers without FXTAS (FXTAS- subgroup, N=7)**

**Spearman correlation coefficient (ρ)**
