# Supplementary material for: Static and dynamic postural control deficits in aging fragile X mental retardation 1 (FMR1) gene premutation carriers
Source: J Neurodev Disord. 2019 Jan 21;11:2. doi: 10.1186/s11689-018-9261-x (PMC6341725; doi:10.1186/s11689-018-9261-x)
Supplement: Supplementary file 2 — Table S2. Correlation coefficients (r) between CGG repeat length and all COP measures and ICARS subscale and total scores of FMR1 premutation carriers with FXTAS (FXTAS+ subgroup, N = 7). (DOCX 15 kb) [file 11689_2018_9261_MOESM2_ESM.docx]

**Additional file 2: Table S2**

| Static stance COP_ML_ standard deviation | r=.49, p=.26 |
| --- | --- |
| Static stance COP_AP_ standard deviation | r=.68, p=.09 |
| Dynamic AP sway COP_ML_ standard deviation | r=.76, p=.05 |
| Dynamic AP sway COP_AP_ standard deviation | r=-.03, p=.95 |
| Dynamic ML sway COP_ML_ standard deviation | r=-.35, p=.44 |
| Dynamic ML sway COP_AP_ standard deviation | r=-.48, p=.27 |
| Static stance COP_ML_ alpha exponent | r=.37, p=.41 |
| Static stance COP_AP_ alpha exponent | r=-.44, p=.32 |
| Dynamic AP sway COP_ML_ alpha exponent | r=.34, p=.46 |
| Dynamic AP sway COP_AP_ alpha exponent | r=-.01, p=.98 |
| Dynamic ML sway COP_ML_ alpha exponent | r=.44, p=.32 |
| Dynamic ML sway COP_AP_ alpha exponent | r=-.58, p=.17 |
| ICARS dysarthria score | ρ =-.36, p=.43 |
| ICARS kinetic score | ρ =-.47, p=.28 |
| ICARS oculomotor score | ρ =.00, p=1.00 |
| ICARS posture & gait score | ρ =-.23, p=.63 |
| ICARS total score | ρ =-.26, p=.57 |

**Table S2. Correlation coefficients (r) between CGG repeat length and all COP measures and ICARS subscale and total scores of FMR1 premutation carriers with FXTAS (FXTAS+ subgroup, N=7)**

**Spearman correlation coefficient (ρ)**
